# Severe Air Pollution and Psychological Distress in China: The Interactive Effects of Coping and Perceived Controllability

**DOI:** 10.3389/fpsyg.2021.601964

**Published:** 2021-06-02

**Authors:** Zhuoying Zhu, Yitong Zhao

**Affiliations:** ^1^Shanghai Mental Health Center, Shanghai Jiao Tong University, Shanghai, China; ^2^Department of Psychology, Wake Forest University, Winston-Salem, NC, United States

**Keywords:** coping, air pollution, depression, anxiety, coping flexibility, trauma

## Abstract

The coping styles of focusing on a stressor (i.e., trauma focus), and moving beyond the emotional impact of a stressor (i.e., forward focus), have both been found beneficial to psychological adjustment. This study investigated whether these two coping styles are similarly associated with adjustment across levels of perceived controllability and beyond European-American contexts. During China’s peak of air pollution in 2014, we surveyed 250 young- to middle- aged adults online to measure their coping behaviors, smog perceptions, and psychological distress, and collected objective data of pollution severity in the respondents’ cities. Results showed that forward-focus coping was generally associated with lower distress and trauma-focus coping was associated with greater distress. Perceived controllability significantly moderated the associations between trauma focus (but not forward focus) and distress. These findings suggest that while forward focus correlated with beneficial adjustment outcomes in coping with air pollution, the extensive processing of event-related cognitions and emotions in trauma focus may be detrimental, especially for events perceived to be less controllable. We discussed the implications of our findings within an interdependent cultural context.

## Introduction

China has gone through rapid industrialization in the past 30 years. This process has brought tremendous economic growth and improved living conditions in the country, but at the same time, series of environmental consequences (World Bank, 1997). Particularly, severe air pollution raised unprecedented public concerns (Kan, 2009). Since 2003, multiple areas in China have been suffering from episodic smog that was observed by citizens (Wei et al., 2009). In December 2013, air pollution reached a historically high level in Eastern China, including Shanghai, Tianjin, and several major provinces – The concentration of atmospheric particulate matter (e.g., PM2.5) increased for months, with a hazardous Air Quality Index (AQI) that exceeded 300 (Zhang, 2013).

Due to its potential of causing myriad health problems, chronical air pollution is an environmental stressor that can be quite challenging for affected public citizens to cope with. Health professionals believe that air pollution can seriously affect respiratory and cardiovascular systems by increasing risks of asthma, respiratory tract infection, COPD, myocardial infarction, and lung cancer (Anderson et al., 1995; Beeson et al., 1998; Peters et al., 2001; Mills et al., 2008; Brook et al., 2010). Pollutants and fine particulates in the air can threaten long-term detrimental physical and psychological health (Wichmann et al., 1989; Samet et al., 2000; Franklin et al., 2006). Physical reactions, including direct respiratory and ophthalmological irritation, trigger the public’s psychological reactions to perceived health threats (Chattopadhyay et al., 1995).

Evidence from animal studies showed that air pollution provoked anxiety, impaired cognition, and depressive-like behaviors (Fonken et al., 2011). In human subjects, air pollution also evoked threat-related reactions, including worry, anxiety, and aggression (Claeson et al., 2013). Increased pollutant concentrations were associated with worsened daily mood (Bullinger, 1989), decreased subjective well-being (Welsch, 2006), and aggravated depression symptoms (Szyszkowicz et al., 2010). These negative psychological states could be strongly associated with pollution levels, even more than somatic symptoms (Lim et al., 2012). This has suggested the importance of investigating people’s psychological processes and coping mechanisms in adjusting to air pollution.

Our study was thus aimed to investigate the psychological correlates of individuals’ adjustment to air pollution during China’s severe smog episodes from January to March 2014. We assessed people’s coping responses, as well as their subjective perceptions of the pollution, in correlating with adjustment (i.e., psychological distress).

### Coping With SMOG: Trauma Focus and Forward Focus

Psychological adjustment is not merely determined by the presence and severity of a stressful or traumatic event (Galatzer-Levy et al., 2012). People vary in their tendencies to adopt various coping strategies during a single stressful encounter (Cox and Ferguson, 1991). The effectiveness of such strategies should also shape adjustment outcomes. Two previously identified coping styles differ in whether people focus on (i.e., trauma focus) or move beyond (i.e., forward focus) the impact of a potentially traumatic event (Bonanno et al., 2011). Given that both coping styles have been found protective following potentially traumatic events in the European-American cultures (Bonanno et al., 2011), it is important to ask whether either or both are effective in the context of coping with severe air pollution, a chronical environmental stressor, beyond European-American contexts.

Classic trauma theories have long emphasized the key component of trauma-focused coping, the full processing, reconstrual, and revisiting of existing traumatic experiences (e.g., Horowitz, 1986). Techniques that encourage addressing, understanding, and integrating traumatic events have been widely used in clinical settings. These techniques, such as emotional journal keeping (Pennebaker, 1997; Smyth, 1998) and exposure treatments (e.g., Foa and Rothbaum, 1998), have been endorsed by professionals as beneficial for individual adjustment.

Forward-focused coping, on the other hand, may also benefit adjustment as it emphasizes maintaining one’s daily activities and goal commitment despite the existing impact of traumatic experiences (Bonanno et al., 2011). Growing empirical studies have shown that cognitions and behaviors that focus on leaving behind the traumatic event, such as distraction, avoidance, optimistic focus on the future, and suppression/repression of emotions, are also associated with adaptive outcomes (for reviews see Taylor and Brown, 1988; Scheier and Carver, 1992, 1993; Bonanno and Kaltman, 1999, 2000; Bonanno, 2004; Westphal et al., 2010).

The distinction between trauma focus and forward focus is similar, but not equivalent to the distinction between the two coping orientations proposed in the dual-process model of coping with bereavement (Stroebe and Schut, 1999). Trauma focus and forward focus somewhat overlap with the model’s key concepts, loss- and restoration orientation (i.e., confronting the death of a loved one vs. adjusting to the life without them; Stroebe and Schut, 1999). However, they apply to contexts that are broader than coping with bereavement. In this study, we resonate with previous arguments about the dual-process model that people may shift between coping styles across time and contexts. We emphasize that adjustment depends on whether an adopted coping style is deemed acceptable and healthy within one’s socio-cultural context (Stroebe and Schut, 2010). Following such reasoning, the evaluation of trauma focus and forward focus’s adaptiveness should also be put into the cultural context where they are used.

### Cultural Perspectives on Coping

Evidence has been sparse in investigating outcomes associated with trauma focus and forward focus within highly interdependent cultural contexts (e.g., East Asian culture). Either coping style may not always be used in culturally appropriate and effective ways. The processing of an event’s significant emotional and cognitive attributes (i.e., trauma focus), for example, may require the receptive listening of a clinical professional or a social partner that can be absent in less resourceful contexts (Flannery, 1990). Attempts to disengage from a traumatic event (i.e., forward focus) may also backfire, if that event signals an ongoing stressor that intensifies over time (Pineles et al., 2011). In this study, we advocate for a culturally sensitive approach in evaluating the two coping styles by supplying evidence within an East Asian culture.

First, we hypothesized that contradictory to findings in European-American samples, trauma focus may be detrimental for East Asians’ adjustment. The “working through” (Horowitz, 1986) of stressful cognitions and emotions involves deliberate mental work that can be exceedingly effortful and demanding (Foa and Kozak, 1986; Brewin, 2003). Recurrent but fruitless reflections on traumatic events increase contact with distressing materials, which may prolong undesirable negative experiences (Stanton et al., 2000; Hanin, 2001). Moreover, aspects of trauma focus (e.g., ruminating over personal loss, negative expressivity) seem incompatible with Eastern cultural values that emphasize relational harmony and socially appropriate emotional inhibition (Markus and Kitayama, 1991; Consedine et al., 2002). Trauma focus may be harmful due to its intensive negative expressivity and emotional burden, particularly in interdependent cultures.

Second, we hypothesize that consistent with evidence in existing studies, forward focus should also be linked to better adjustment in East Asians. Instead of dwelling on one’s negative past, people who cope with a forward focus engage in external activities outside the stressful context, which should promote enacted social support during times of need and positive future expectancies. Such activities seem to be encouraged by the cultural norms deeply rooted in Confucian and Taoist ideologies in East Asian cultures (Hodges and Oei, 2007; Yang et al., 2016). Therefore, forward focus should benefit adjustment, as it helps maintain individuals’ daily goal commitment and social engagement, in addition to preventing excessive exposure to the stressor.

In this study, we selected severe air pollution as a collective stressor, and expect the two coping styles to differentially correlate with adjustment outcomes in a sample of Chinese participants. Trauma focus should be associated with worse adjustment (i.e., higher psychological distress), while Forward focus should be associated with lower distress. In addition, we will test the moderating role of perceived situational controllability, which typically predict both coping types and outcomes (Conway and Terry, 1992; O’Connor and Shimizu, 2002). Trauma focus may be particularly harmful to adjustment, when individuals lack perceived control over the stressful pollution conditions. Correspondingly, we hypothesize that perceived controllability will attenuate the positive association between trauma focus and psychological distress. We did not make strong hypotheses about the moderation of perceived controllability on the association between forward focus and adjustment.

## Materials and Methods

### Participants and Procedures

Sample size was determined in advance using G^∗^Power 3.1.9.2 (Faul et al., 2007). To our knowledge, no existing research has shown the effect size of an interaction between trauma focus and controllability. Therefore, we used the eta square (0.043) for the effect of coping ability type on complicated grief, another adjustment outcome in the presence of trauma and loss (Burton et al., 2012). Using the formula f square = eta square/(1-eta square), a small effect size (f square = 0.045) and a type I error rate of 0.05 were plugged in the program to reach a desired power of 0.80. The recommended sample size was 218 to find a significant effect of the two coping variables in an *F* test for multiple regression models.

An internet survey method was used, aiming to reach a sample of 250 participants. Online questionnaires measuring coping, appraisals, and psychological distress were distributed on *Guokr*^1^, a leading popular science Chinese website. Before proceeding to the survey, participants were informed of the study’s purpose and procedures, data confidentiality, and their freedom to withdraw at any time. Then, they gave online consent and proceeded to the actual survey.

The survey was launched in the peak of China’s air pollution on January 5th, 2014. Recruitment advertisements were posted under a special section for social sciences surveys and on one of the most popular social media platforms, *Sina Weibo*. Data collection was completed by the end of March 2014. Adult participants were given of a brief summary of the smog status in China during that time and reported how they subjectively perceive and objectively coped with the situation. They also answered questions about demographics, perceived social support, and adjustment outcomes. All measures were in Chinese after being translated and back-translated to ensure validity.

The final sample size was 209 after excluding participants who were younger than 18 years old and/or finished the survey in extremely short time (i.e., less than 4 minutes). All participants were Chinese citizens located across 49 urban cities, most of which were greatly impacted by smog (e.g., 29% Shanghai, 24% Beijing). The majority (80%) were young- to middle- aged adults between 20 and 40 years old (*Mean* = 29.15, *SD* = 8.58), 56% females, and 49% married and 44% single. Participants had relatively higher socioeconomic standing (see Table 1 for details of income, education, and employment status). Over a third (34%) had a family income over 100,000 Yuan, 86% with at least a bachelor’s degree, and 72% were employed when they participated in the study. The data that support the findings of this study are openly available in Open Science Framework at https://osf.io/cfh6r/.

**TABLE 1:** Participants’ distributions in socioeconomic variables.

Variable	Level	Percentage (%)
Marital status	Single	44.0
	Married	49.3
	Cohabitating	1.9
	Bereaved	0.0
	Divorced	2.4
	Separated	0.0
	Other	2.4
Family income	Less than 10,000	10.5
	10,000 – 19,999	11.0
	20,000 – 39,999	16.3
	39,999 – 69,999	14.4
	69,999 – 99,999	13.9
	More than 100,000	34.0
Education level	None	0.5
	Elementary school and below	1.4
	Elementary school	1.0
	Middle School	3.8
	High school / technical secondary school	7.7
	Bachelor / Junior college	60.8
	Master and above	24.9
Employment	Employed	71.8
	Students	22.0
	Unemployed	5.3
	Retired	1.0

### Measures

#### Coping

The Perceived Ability of Coping with Trauma (PACT; Bonanno et al., 2011) was adapted to assess *actual* behaviors in coping with smog. The PACT originally measures participants’ perceived abilities to utilize different coping styles facing traumas. In this study, participants were presented with a list of strategies or coping behaviors from the original scale. Then, they rated the extent to which they have engaged in each behavior since the occurrence of smog. The PACT assesses trauma focus and forward focus, were both rated on a 7-point Likert scale (1 = “never,” 7 = “always”). The 12-item *forward focus* subscale captures coping abilities associated with moving beyond traumatic experience (e.g., “Distract myself to keep from thinking about the event”). The 8-item *trauma focus* subscale captures abilities to fully experience the cognitive and emotional significance of the stressor (e.g., “Remember the details of the event”). They both demonstrated acceptable to good reliabilities in the United States (forward focus: *α* = 0.85, trauma focus: *α* = 0.79; Bonanno et al., 2011) and in China (forward focus: *α* = 0.92, trauma focus: *α* = 0.67 with one item removed; Burton et al., 2012). Scores were obtained by averaging the subscales, which had good reliabilities (*α* = 0.90 for forward focus, *α* = 0.73 for trauma focus).

#### Psychological Distress

Two indicators of psychological distress, depression and anxiety [*r*(207) = 0.79, *p* < 0.001], were averaged to create a composite score (*α* = 0.88) after standardizing. Depression (*α* = 0.87) was measured by 11 items from the Center for Epidemiologic Studies-Depression Scale (CES-D; Radloff, 1991). Items (e.g., “I felt depressed”; “I felt that people dislike me”) were rated on a 3-point Likert scale (1 = “almost never or never,” 3 = “often or always”). Anxiety (*α* = 0.90) was measured by the Generalized Anxiety Disorder 7-item (GAD-7) scale (Spitzer et al., 2006), rated on a 4-point scale (1 = “not at all sure,” 4 = “nearly every day”). Both indicators were summed scores of the full scale.

#### Controllability

Individuals’ subjective appraisals of smog were measured by the Stress Appraisal Measure (SAM; Peacock and Wong, 1990), rated on a 5-point Likert scale (1 = “completely disagree,” 5 = “completely agree”). This measure assesses participants’ perceptions towards the smog situation using three 4-item subscales, *Controllable-by-self* (*α* = 0.83; e.g., “have ability to do well”), *Uncontrollable* (*α* = 0.83; e.g., “beyond anyone’s power”), and *Controllable-by-others* (*α* = 0.45; e.g., “someone I can turn to”), all related to our construct of interest, appraised situational controllability.

Confirmatory Factor Analyses (CFA) on the three subscales were conducted for two reasons. First, SAM has not been validated in Chinese cultural contexts. Second, contents of all three subscales seem relevant to the number of resources, help, or skills perceived available in coping with smog. *Controllable-by-others* especially applies to the current environmental stressor that is collectively faced by residents in concerned areas and affected by actions of the government and large corporations. After eliminating 3 items with unsatisfactory loadings (see CFA results and reasoning in Table 2), a single composite of Controllability was finally calculated by averaging scores of the rest 9 items (*α* = 0.84).

**TABLE 2:** CFA factor loadings of items measuring controllability.

	CFA: Three-factor structure			CFA: One-factor structure (all 12 items)	CFA: One-factor structure (9 items)
			
Item	Controllable-by-self	Uncontrollable	Controllable-by-others	Controllability	Controllability
1. Have ability to do well	0.78			0.58	0.59
2. Have what it takes	0.72			0.44	0.45
3. Will overcome problem	0.93			0.67	0.69
4. Have skills necessary	0.62			0.76	0.77
13. Totally hopeless		0.77		−0.73	−0.72
14. Outcome uncontrollable		0.88		−0.58	−0.58
15. Beyond anyone’s power		0.49		−0.69	−0.69
16. Problem unresolvable		0.87		−0.65	−0.64
17. Someone I can turn to			0.45	0.29	
18. Help available			0.85	0.33	
19. Resources available			0.42	0.50	0.47
20. Anyone who can help			0.18	−0.35	
Cronbach’s alpha	0.83	0.83	0.45	0.84	0.84

*Promax rotation was used for the three-factor structure. Item 1–4: Controllable-by-self; Item 13–16: Uncontrollable; Item 17–20: Controllable-by-other. Item 13–16 were reverse-keyed in calculating internal consistencies of the single factor, Controllability. For a one-factor structure, item 1–4 positively loaded on the first factor (named Controllability) with loadings greater than 0.44. Expectedly, items from Uncontrollable all had negative loadings greater than 0.58. Item 17, 18, and 20 from Controllable-by-Other had loadings below.40, leaving only item 19 with satisfactory loading (i.e., “resources available,” loading = 0.50). Controllability was finally calculated by averaging scores of the 9 items, given their satisfactory loadings (i.e., loadings of item 2 and 19 larger than 0.45, the rest larger than 0.58). This composite also had slightly less Skewness (0.21) and Kurtosis (0.01) compared to averaged scores of single subscales, Uncontrollable (Skewness = −0.26, Kurtosis = −0.49) and Controllable-by-Self (Skewness = 0.27, Kurtosis = −0.41).*

#### Social Support

Perceived social support (*α* = 0.86) was measured by the Perceived Social Support Scale (PSSS; Lu et al., 2011), rated on a 7-point Likert scale (1 = “Strongly disagree,” 7 = “Strongly agree”). Participants rated items that describe the level of social support they got since smog arose. The scale consists of 4 items measuring both the perceived quantity and quality of received emotional support (e.g., “I think I had enough emotional support”) and instrumental support (e.g., “I think I had enough financial support”). Support resources may have buffered the adverse psychological effects of smog (Bonanno et al., 2004), and was thus measured as a control.

#### City Pollution Severity

Participants’ locations during survey completion were recorded based on IP addresses. Based on their locations, the real-time data of air pollution severity was obtained from China’s National Realtime Publishing Platform for Daily Air Quality. This system delivered real-time data on concentrations of ambient air pollutants, measured by state-controlled air-monitoring stations. We included three objective indicators of city pollution severity, PM2.5 concentration, O3 concentration, and the average death rate of respiratory diseases. These three indicators reflect different negative consequences of pollution, but may not be directly correlated in a certain direction (Chen et al., 2019; Lei et al., 2019). Scores were all averaged across the 3-month data collection period. Higher scores indicate greater severity.

#### Perceived Health Change

Given the somatic expression of distress evident in Chinese culture (Chang et al., 2005), the extent to which participants perceived their health worsened due to air pollution was measured using one item, “How have your health conditions changed after smog?,” rated on a 3-point scale (1 = “better than before,” 2 = “no change,” 3 = “worse than before”). Many participants (56%) reported worsened health, and none reported improved health after smog.

## Results

### Zero-Order Correlations

Table 3 presents the descriptives and correlations between variables. We were right to have planned to control for age, which was negatively correlated with distress [*r*(204) = −0.18, *p* < 0.05], and perceived health change, which was correlated with distress [*r*(207) = 0.29, *p* < 0.01]. None of the objective indicators of city pollution severity were significantly correlated with distress, while individuals’ perceived controllability [*r*(207) = −0.32, *p* < 0.01] and support were both negatively correlated with distress [*r*(207) = −0.37, *p* < 0.01]. These controls will all be included in regression models.

**TABLE 3:** Means, standard deviations, and zero-order correlations.

	Variable	Mean	*SD*	1	2	3	4	5	6	7	8	9	10	11
1	Age	29.45	8.24											
2	City PM2.5 concentration	90.01	31.19	−0.14*										
3	City O3 concentration	59.94	11.00	0.27**	−0.72**									
4	City respiratory disease death rate	10.82	6.22	0.26**	−0.14*	0.12								
5	Perceived health change	2.56	0.51	0.08	0.04	−0.02	−0.05							
6	Trauma focus	3.65	1.02	−0.07	0.01	−0.03	−0.08	0.43**						
7	Forward focus	4.34	1.02	0.04	−0.1	0.08	−0.03	0.12	0.42**					
8	Support	4.38	1.4	0.08	0.02	−0.06	0.07	−0.15*	−0.09	0.30**				
9	Controllability	2.46	0.74	−0.02	−0.18*	0.08	−0.03	−0.37**	−0.22**	0.20**	0.24**			
10	Depression	18.54	4.29	−0.23**	0.01	0.01	−0.17*	0.27**	0.37**	−0.11	−0.40**	−0.29**		
11	Anxiety	12.67	4.12	−0.1	−0.05	−0.01	−0.09	0.29**	0.35**	−0.12	−0.31**	−0.32**	0.79**	
12	Emotional distress	0	0.94	−0.18*	−0.02	0.00	−0.14	0.29**	0.38**	−0.12	−0.37**	−0.32**	0.94**	0.94**

N = 206 for age. Missing data was also present for city pollution severity indicators (PM2.5: N = 202, O3: N = 198, Respiratory: N = 201). N = 209 for other variables. Trauma focus, Forward focus, and Support are rated on a scale from 1 to 7. Perceived health change is rated on a scale from 1 to 3. Controllability and the is rated on a scale from 1 to 5. Emotional distress is the main outcome variable averaged from standardized scores of depression and anxiety, and thus has a mean of 0. *p < 0.05. **p < 0.01.

Our hypothesis that trauma focus may harm adjustment was supported by a moderate positive correlation between trauma focus and distress [*r*(207) = 0.38, *p* < 0.01]. Forward focus was marginally correlated with less distress [*r*(207) = −0.12, *p* = 0.08]. Additionally, correlations between coping styles and controllability suggest that people who perceived the situation as less controllable had more tendencies to focus on [*r*(207) = −0.20, *p* < 0.01)], rather than move past the emotional impact of smog [*r*(207) = 0.22, *p* < 0.01]. Next, controls were entered in the first step of hierarchical regression. In step 2, two coping variables were simultaneously entered. In step 3, controllability and its interaction with the two coping variables were entered to test the moderation hypothesis.

### Hierarchical Linear Regressions

Table 4 presents the standardized effects of all key variables in predicting distress. All controls, but not city pollution severity indictors, significantly predicted distress in step 1 (*R*^2^ = 0.23, Δ*F*(6, 187) = 9.20, *p* < 0.001). In step 2, their effects attenuated when two coping variables were included (*R*^2^ = 0.32, Δ*F*(2, 185) = 12.22, *p* < 0.001). Coping styles additionally explained 9% of the outcome variability. Supporting our first hypothesis, trauma focus (*b* = 0.33, *95% CI* = [0.20, 0.47], β = 0.36, *t*(185) = 4.85, *p* < 0.001). Our second hypothesis was also supported that forward focus significantly predicted less distress (*b* = −0.19, *95% CI* = [−0.32, −0.06], β = −0.20, *t*(185) = −2.87, *p* < 0.01) in step 2. However, forward focus had a non-effect on distress in step 3 (*b* = −0.004, *95% CI* = [−0.38, 0.37], β = −0.005, *t*(182) = 0.11, *p* = 0.91), after including controllability and the two interaction terms in the model. Trauma focus’s effect remained significant and became larger (*b* = 0.67, *95% CI* = [0.32, 1.02], β = 0.73, *t*(182) = 3.76, *p* < 0.001) after allowing for the moderations of controllability. Supporting our third hypothesis, controllability significantly moderated trauma focus’s effect (*b* = −0.15, *95% CI* = [−0.28, −0.01], β = −0.52, *t*(182) = −2.12, *p* < 0.05), but not forward focus’s effect (*b* = −0.05, *95% CI* = [−0.19, 0.09], β = −0.25, *t*(182) = −0.71, *p* = 0.48). Significant R square changes from step 2 to step 3 indicate a significant increase in proportion of variability explained in distress (*R*^2^ = 0.35, Δ*F*(3, 182) = 2.93, *p* < 0.05).

**TABLE 4:** Hierarchical linear regressions.

Predictors	Step 1	Step 2	Step 3
*Controls*			
Age	−0.16*	−0.13*	−0.14^†^
Perceived health change	0.26***	0.13^†^	0.10
Social support	−0.31***	−0.2***	−0.25***
City PM2.5 severity	−0.02	−0.03	−0.09
City O3 severity	0.02	0.04	0.01^†^
City respiratory disease death rate	−0.06	−0.05	−0.07
*Coping*			
Trauma focus		0.36***	0.73***
Forward focus		−0.20**	0.004
*Controllability and interactions*			
Controllability			0.45
Trauma focus × Controllability			−0.52*
Forward focus × Controllability			−0.25
*R*^2^	0.23	0.32	0.35
Δ*R*^2^	0.24	0.09	0.03
Δ*F*	9.20***	12.22***	2.93*

Coefficients were standardized betas in predicting psychological distress. Without including control variables, the model using trauma focus, controllability, and their interaction to predict distress was also significant (R^2^ = 0.22, ΔF(3,205) = 19.43, p < 0.001). The interaction between trauma focus and controllability also remained similar to when covariates were included, b = −0.15, 95% CI = [−0.29, −0.02], β = −0.54, t(205) = −2.19, p = 0.03. ^†^p < 0.10; *p < 0.05; *p < 0.01; ***p < 0.001.

**FIGURE 1:**
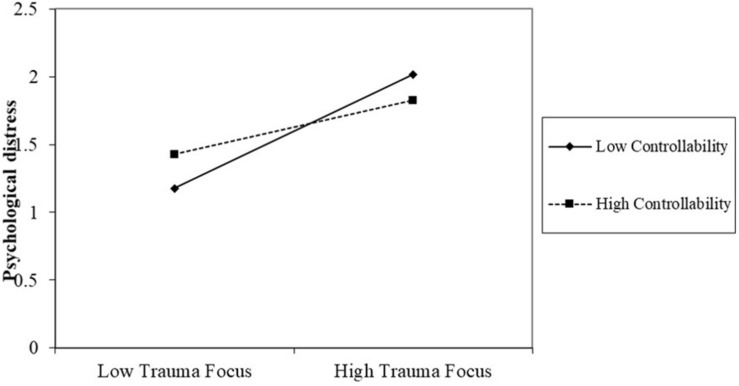
The interaction between trauma focus and controllability.

**FIGURE 2:**
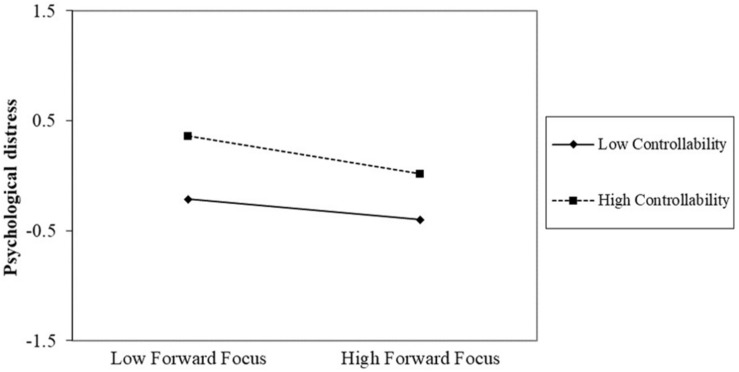
The interaction between forward focus and controllability.

The relationships between coping and distress were plotted in Figure 1 (trauma focus) and Figure 2 (forward focus) at higher and lower levels of controllability (Aiken and West, 1991), holding all controls constant. Simple Slopes showed that at higher controllability (*M*+*SD* = 3.20), the effect of trauma focus was smaller [*b* = 0.20, *t*(182) = 2.20, *p* < 0.05] than its effect [*b* = 0.41, *t*(182) = 5.07, *p* < 0.001] at lower controllability (*M-SD* = 1.73), both significant. Despite a non-significant interaction between forward focus and controllability, the positive effect of forward focus was significant only at high [*b* = −0.17, *t*(182) = −1.98, *p* < 0.05] but not low controllability (*b* = −0.09, *t*(182) = −1.07, *p* = 0.29).

## Discussion

The present research investigated the effects of trauma-focused and forward-focused coping on psychological distress when coping with air pollution in Chinese culture. Consistent with our hypothesis, trauma focus was linked to more affective symptoms (i.e., depression and anxiety) that are indicative of worse adjustment, while forward focus was not significantly linked to adjustment. Our study also provides initial evidence on the moderation of perceived controllability in the relationship between the two coping styles and adjustment. Controllability attenuated the positive effect of trauma focus on distress, such that trauma focus is more strongly associated with distress when individuals perceived smog as *less controllable*. This interaction remained significant even controlling for age, objective pollution severity, and perceptions of health change due to smog and social support.

Inconsistent with previously found adaptive effects ofboth coping styles (e.g., Bonanno et al., 2011), the main effects ofcoping showed that trauma focus may be maladaptive in a highlyinterdependent culture. Trauma focus positively correlated withdistress during severe air pollution, while forward focus showednon-effects in the current sample. Such discrepancies might beexplained by the characteristics of Eastern cultural values. East Asians emphasize interpersonal relatedness (Markus and Kitayama, 1991) instead of individual preferences and internal states (Russell and Yik, 1996). They more readily recognize the feelings and attitudes of others (Varnum et al., 2010) and prioritize others’ needs over personal goals. Therefore, the negative expressivity in trauma focus could undermine support resources (Matsumoto, 1990; Forest et al., 2014; Senft et al., 2020) and worsen expressors’ personal image (i.e., judged as highly emotional; He and Zhang, 2011). These potential social costs may explain trauma focus’s harmful associations with worse adjustment in China.

Forward focus did not seem beneficial on its own (i.e., without controlling for controllability and its moderation). This is potentially because unlike other collective stressors or trauma, air pollution in China was not a discrete event. Given that the situation was chronically uncertain with a lack of foreseeable measures, people may have needed to shift their coping responses in meeting the changing situational demands (Moos and Swindle, 1990; Rivkin and Taylor, 1999). Therefore, we only found limited benefits of forward focus in this study.

Notably, individuals’ subjective perception of situational controllability, but not the objective severity of the stressor (e.g., pollutant concentration), significantly correlated with adjustment. Descriptives showed that smog was perceived as generally uncontrollable (i.e., ratings were higher than scale midpoints), and that the overall sample tended to adopt a forward-focused, instead of trauma-focused coping style. Moderation and simple slopes analyses additionally supported that trauma focus was detrimental especially under *low* controllability, while forward focus significantly correlated with lower distress only under *high* controllability. Together, these findings suggest that in uncontrollable situations, individuals may fare better if they did not focus on but rather moved past its perceived negative impact. One caveat in explaining this result is that the current data captured a potentially limited variation in controllability due to a relatively low sample mean. Thus, the link between forward focus and better adjustment may have been underestimated without including stressful contexts of generally higher controllability.

Finally, even though controllability positively correlated with forward focus and negatively correlated with trauma focus, these two coping variables were positively correlated themselves. This shows that participants may have adopted multiple responses in coping with smog. The concept of Coping Flexibility (Cheng, 2001; Bonanno, 2004, 2005) emphasizes the flexible employment of multiple strategies without heavily relying on one coping style. Our moderation results were also consistent with the idea of *strategy-situation fit* in the Coping Flexibility Hypothesis (Kato, 2012; see also Cheng et al., 2014), which suggests that no single strategy is inherently (mal)adaptive, but that its effectiveness should be evaluated based on the contexts where it is used. For instance, strategies that merely modify the emotional response to a stressor (e.g., cognitive reappraisal) can be ineffective in *highly controllable* situations (Zakowski et al., 2001; Troy et al., 2013). Our moderation patterns (i.e., controllability attenuated the harmful effects of trauma focus), by contrast, suggest a different perspective. It seems that strategies that focus on processing event-related emotions and cognitions may backfire in *uncontrollable* situations, where effortful reconstruals and exposure to distressing materials can be futile and only lead to gloomy prospects (e.g., by constantly reminding oneself “I can’t change anything”).

### Limitations and Future Directions

The current research has several limitations. First, we were interested in the causal effects of coping, but only retrospective reports of coping behaviors were obtained in predicting concurrent outcomes. Future research could adopt longitudinal approaches (e.g., experience sampling) to allow for within-person variation in the use of coping styles and their long-term consequences. Second, characteristics of both the interdependent cultural context and the uncontrollable smog situation may have contributed to the inconsistencies between prior literature (i.e., both coping styles are beneficial) and our results (i.e., trauma focus is detrimental). Future studies could replicate our findings in other interdependent cultures and in situations of varying levels of controllability. Each coping response and each situational characteristic could be measured and matched to generate a full profile of their context-specific adaptiveness (Cheng, 2001; Aldao et al., 2015). Finally, the potential costs associated with processing uncontrollable stressors may exist across cultures, regardless of the degree of interdependence (e.g., use of rumination; Afifi et al., 2013; unsuccessful cognitive reappraisal; Ford and Troy, 2019). These costs may be particularly likely to occur, if individuals are inflexible in disengaging from overwhelming negative emotions and self-focused cognitions (De Lissnyder et al., 2012). A lack of perceived control might even produce more intense distress in highly independent cultures, where personal autonomy is greatly valued (Bernardi and Jobson, 2019). Future cross-cultural research should test such ideas and inform clinical practices in treating trauma-related symptoms and emotion dysregulation.

## Data Availability Statement

The datasets presented in this study can be found in online repositories. The names of the repository/repositories and accession number(s) can be found below: https://osf.io/cfh6r/.

## Ethics Statement

The studies involving human participants were reviewed and approved by Teachers College at Columbia University Institutional Review Board. The ethics committee waived the requirement of written informed consent for participation.

## Author Contributions

ZZ designed the study and contributed to the collection of data. ZZ and YZ performed data analyses. Both authors drafted the manuscript and provided critical revisions.

## Conflict of Interest

The authors declare that the research was conducted in the absence of any commercial or financial relationships that could be construed as a potential conflict of interest.

## Footnotes

1 https://m.guokr.com/
